# Caffeic acid phenethyl ester is protective in experimental ulcerative colitis via reduction in levels of pro-inflammatory mediators and enhancement of epithelial barrier function

**DOI:** 10.1007/s10787-017-0364-x

**Published:** 2017-05-20

**Authors:** Mohammed N. Khan, Majella E. Lane, Paul A. McCarron, Murtaza M. Tambuwala

**Affiliations:** 1 SAAD Centre for Pharmacy and Diabetes, School of Pharmacy and Pharmaceutical Science, Ulster University, Coleraine, County Londonderry BT52 1SA Northern Ireland, UK; 2 UCL School of Pharmacy, 29-39 Brunswick Square, London, WC1N 1AX UK

**Keywords:** Inflammatory bowel diseases, Colitis, Natural, Nuclear factor kappa beta, Pro-inflammatory cytokines, Intestinal permeability

## Abstract

**Background:**

Inhibition of the nuclear factor *kappa beta* (NF-κβ) pathway has been proposed as a therapeutic target due to its key role in the expression of pro-inflammatory genes, including pro-inflammatory cytokines, chemokines, and adhesion molecules. Caffeic acid phenethyl ester (CAPE) is a naturally occurring anti-inflammatory agent, found in propolis, and has been reported as a specific inhibitor of NF-κβ. However, the impact of CAPE on levels of myeloperoxidases (MPO) and pro-inflammatory cytokines during inflammation is not clear. The aims of this study were to investigate the protective efficacy of CAPE in the mouse model of colitis and determine its effect on MPO activity, pro-inflammatory cytokines levels, and intestinal permeability.

**Method:**

Dextran sulphate sodium was administered in drinking water to induce colitis in C57/BL6 mice before treatment with intraperitoneal administration of CAPE (30 mg kg^−1^ day^−1^). Disease activity index (DAI) score, colon length and tissue histology levels of MPO, pro-inflammatory cytokines, and intestinal permeability were observed.

**Results:**

CAPE-treated mice had lower DAI and tissue inflammation scores, with improved epithelial barrier protection and significant reduction in the level of MPO and pro-inflammatory cytokines.

**Conclusion:**

Our results show that CAPE is effective in suppressing inflammation-triggered MPO activity and pro-inflammatory cytokines production while enhancing epithelial barrier function in experimental colitis. Thus, we conclude that CAPE could be a potential therapeutic agent for further clinical investigations for treatment of inflammatory bowel diseases in humans.

**Electronic supplementary material:**

The online version of this article (doi:10.1007/s10787-017-0364-x) contains supplementary material, which is available to authorized users.

## Introduction

Inflammatory bowel disease (IBD) is an idiopathic disorder, generally categorised as either Crohn’s disease (CD) or ulcerative colitis (UC) (Neurath 2014; Ford et al. 2011). There is no therapeutic cure for IBD and the current disease management strategies possess several drawbacks. For example, immunomodulatory agents, such as azathioprine and 6-mercaptopurine, cause bone marrow depletion and damage to both white blood cell and hepatic cell populations. Furthermore, results from recent clinical trials confirm that azathioprine is ineffective in UC (Ardizzone et al. 2006; Kamath et al. 2016; O’Connor et al. 2010) and sulfasalazine causes ruptures in liver tissue and decreases platelets count in blood (de Abajo et al. 2004; Rubin 1994). Furthermore, pulmonary disorders are reported in IBD patients treated with chimeric monoclonal antibodies, such as infliximab (Patel et al. 2016).

The clinical symptoms of IBD range from episodes of relapse and remission with mild inflammation and discomfort to a chronic ulcerative disease requiring surgical removal of the inflamed gut. The current therapeutic strategies for IBD are generally limited, but recent clinical advancement has occurred in immunotherapy using monoclonal antibodies. This approach is directed against inflammatory mediators, such as TNF-α (Targan 2006; Subramanian et al. 2017; Gecse and Lakatos 2017; Chan and Ng 2017). However, these biological agents are expensive and result in severe side effects and life threatening complications (Cote-Daigneault et al. 2015; Blonski and Lichtenstein 2006; Clarke and Regueiro 2012; Cohen and Thomas 2006). Hence, there is a need in the field of IBD therapy to develop new therapeutics, which are effective, safe, and economical. One way to achieve this is investigation into the anti-inflammatory effect of natural compounds and understanding their mechanism of action. Lack of specificity and the encumbrance of severe side effects necessitate further investigation into effective and safer options for treating IBD (Pichai and Ferguson 2012), which is the aim of this current study.

Colonic specimens from UC patients display overexpression of transcription factor nuclear factor kappa beta (NF-κβ) (Atreya et al. 2008). NF-κβ is up regulated by TNF-α, interleukin (IL), interferon, chemokines, and DNA damaging agents during the inflammatory phase. Similar effects are observed following exposure to lipopolysaccharide derived from bacterial cell wall components (Lawrence 2009b; Xavier and Podolsky 2007). In UC, levels of inflammatory mediators, such as TNF-α, interleukins, and interferons, increase due to the over stimulation of NF-κβ during inflammation (Schreiber et al. 1998). It is feasible, therefore, that inhibition of NF-κβ may be of therapeutic benefit in UC, which forms the hypothesis of our current work.

**Fig. 1:**
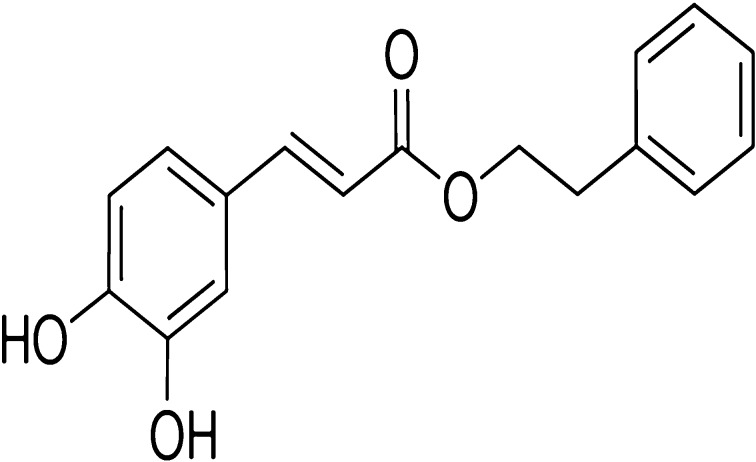
Chemical structure of caffeic acid phenethyl ester (CAPE)

Novel pharmacological inhibitors of NF-κβ are currently available, but these compounds inflect toxicity and severe side effects in humans. Hence, we have selected caffeic acid phenethyl ester (CAPE), a phenolic constituent derived from honeybee propolis and shown in Fig. 1, for further study. It possesses no known adverse side effects (Tolba et al. 2014; Liao et al. 2003). CAPE possesses potent anti-inflammatory properties, which are attributed to its selective inhibition of NF-κβ. Recently, CAPE has been reported to inhibit other relevant pathways, such as MAPK and PI3K (Natarajan et al. 1996; Ozturk et al. 2012; Lin et al. 2013; Pramanik et al. 2013; Cho et al. 2014). CAPE represses translocation of NF-κβ, either by inhibition of Iκβ degradation or by blocking of NF-κβ and DNA binding (Wang et al. 2010; Bezerra et al. 2012). It has been reported that inflammatory markers, such as INF-ϒ, IL-6, IL-β, TNF-α, and IL-10, cause degradation of Iκβ, which results in induced overexpression of NF-κβ (Lang et al. 2004). CAPE inhibits this overexpression of NF-κβ via prevention of degradation of Iκβ (Wang et al. 2010; Lee et al. 2008).

The most recent evaluation of the anti-inflammatory activity of CAPE has been limited to either cell culture models or rat models of 2,4,6-trinitrobenzenesulfonic acid-peptidoglycan polysaccharide-induced UC (Armutcu et al. 2015; Ek et al. 2008; Fitzpatrick et al. 2001; Kim et al. 2013). However, the pathophysiological mechanism, such as macroscopic, microscopic changes in the colon and effect on pro-inflammatory cytokine levels, and mucosal barrier function, by which CAPE exerts its anti-inflammatory activity, have not been fully explored (Fitzpatrick et al. 2001; Michaluart et al. 1999; Ek et al. 2008; Cooper et al. 1993). Hence, the activity of CAPE has not been studied in relevant in vivo models, which are physiologically more representative of the human disease (Chassaing et al. 2014). Thus, the main aspect of this study, which differentiates it from the previous work, is assessment of the activity of CAPE on the colon at macroscopic and microscopic levels, its effect on MPO and pro-inflammatory cytokine levels and altered mucosal permeability in a mouse model of colitis, which is physiologically relevant to human disease (Tambuwala et al. 2015). The findings of this study will provide an insight into the anti-inflammatory efficacy of CAPE during colitis in terms of changes in the levels of the disease activity index (DAI) score, colon MPO, pro-inflammatory cytokines, and epithelial barrier function.

## Materials and methods

### Materials

Caffeic acid phenethyl ester (97%) was purchased from Sigma-Aldrich Ltd. (Dorset, UK) and dextran sodium sulphate (DSS) was procured from MP Biomedicals (Bedford, UK) (molecular weight 36,000–50,000). CAPE was administered by IP injection at a dose of 30 mg kg^−1^ on a daily basis for 7 days. The injection was prepared by dissolving CAPE (1.0 mg) in 1.0 ml of sterile aqueous solution containing 25% PEG 200.

### Dextran sodium sulphate model of induced colitis

For DSS colitis-induced experiments, 12-week-old C57BL/6 female mice were used (Charles River, UK). The Ulster University Animal Research Ethics Committee and UK Home Office approved all procedures described, under Project license (PL2768).

Colitis was induced by administering 2.5% w/v DSS in drinking water over a period of 7 days. The DAI score was used to record morphological changes, such as weight loss, stool consistency, and presence of blood in faeces. On termination of the experiment, mice were sacrificed by cervical dislocation (Egger et al. 2000; Okayasu et al. 1990). The isolated colon was excised, washed in PBS, and laid flat on moist tissue to measure its length. Sections, approximately 1.0 cm, of excised colonic tissue were fixed in 10% paraformaldehyde (pH 7.4; phosphate-buffered saline) and embedded in paraffin. Sections (4 μm) were cut and stained with hematoxylin and eosin. Histologic assessment and scoring of colon tissue sections were carried out in a blinded fashion based on previously defined parameters (Sutherland et al. 1987). All tissue slides were imaged using light a microscopy at 5× and 10× magnifications.

### Colon cytokine and myeloperoxidases measurements

Post-mortem colon tissue was homogenised using a method adapted from processing lung tissue (Mangan et al. 2006). Levels of pro-inflammatory cytokines, such as INF-γ, IL-6, IL1-β, TNF-α, and IL-10, were detected using V-Plex Assay Plates (Meso Scale Diagnostics; Rockville, MD, USA) and assayed as per the manufacturer’s protocol. MPO activity was detected using *o*-phenylenediamine dihydrochloride as substrate and the data were interpolated from an MPO standard curve (Sigma). Levels of cytokines and MPO were expressed as pg per mg or U per mg, respectively, relative to colon protein (Cummins et al. 2008).

### Assessment of NF-κβ activation in colon tissue

Colon tissue was homogenised and lysate then analysed for NF-κβ/p65 levels using a Nuclear Extraction kit (ActiveMotif, Carlsbad, USA) in accordance with the manufacturer’s protocol (Lin et al. 2014).

### In vivo intestinal permeability measurements

Mice were exposed to 7 days of DSS treatment, which was followed by standard oral gavage of fluorescein isothiocyanate (FITC)-labelled dextran (4 kDa) at a dose of 0.6 mg g^−1^ of body weight. Mice were euthanised 4 h later and blood removed by cardiac puncture. Plasma was separated and FITC levels in plasma determined by fluorometry (Tambuwala et al. 2010).

### Statistical analysis

Results were expressed as mean ± standard error of the mean (SEM) for a series of experiments. Data were assumed to be normally distributed and statistical analyses were carried out using Prizm GraphPad V6 software (GraphPad, San Diego, CA, USA). A paired *t* test was used for comparisons of paired treatments between two groups, unpaired *t* tests for comparisons of unpaired treatments between two groups, and one-way ANOVA using Bonferroni multiple comparisons tests for treatments of three groups or more. *P* values ≤0.05 were considered to be significant.

### Ethical considerations

The Ulster University Animal Research Ethics Committee and UK Home Office approved all procedures described, under Project license (PL2768). Severity levels were graded as mild by the UK Home Office.

## Results

### CAPE ameliorates disease in DSS-induced colitis

**Fig. 2:**
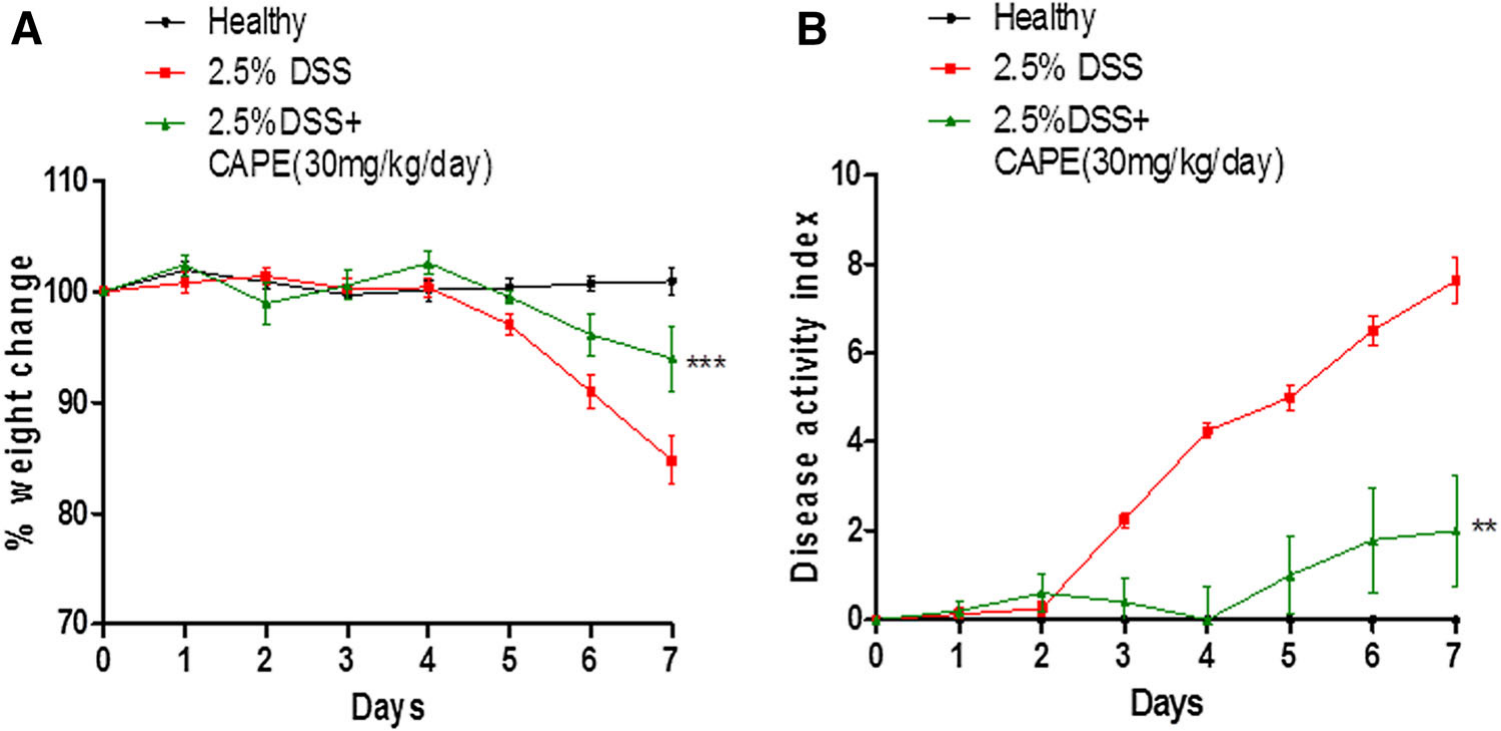
Lowered percentage weight loss and DAI score in mice treated with CAPE during DSS-induced colitis. **a** Percentage weight loss was assessed in mice treated with DSS-alone (*red line*), DSS and CAPE (*green line*), and no DSS healthy mice (*black line*). **b** Disease activity index was assessed in mice treated with DSS-alone (*red line*), DSS and CAPE (*green line*), and no DSS healthy mice (*black line*) over 7 days. Each control and experimental group contained a minimum of 5–6 individual mice (*P* < 0.001–0.01)

It has been reported by us and several researchers that colitis is a collection of symptoms, such as weight loss, diarrhoea, and blood in faeces, collectively described by the DAI and shortening of colon length (Ogawa et al. 2004; Taghipour et al. 2016; Chassaing et al. 2014; Chen et al. 2007). To study the protective effect of CAPE on mice with DSS-induced colitis, we recorded the weight of each mouse in all groups daily for 7 days. Figure 2a shows significantly (*P* < 0.001) lowered weight loss in DSS + CAPE-treated mice when compared to the DSS-only group. Similarly, Fig. 2b shows that mice in the DSS-alone group had the highest DAI score, which confirmed the development of colitis. Mice treated with CAPE showed a significantly (*P* < 0.01) lower DAI score, when comparison is made to the DSS-only group. This finding suggests that CAPE was protecting mice against weight loss and the occurrence of diarrhoea and appearance of blood in faeces during DSS-induced colitis.

**Fig. 3:**
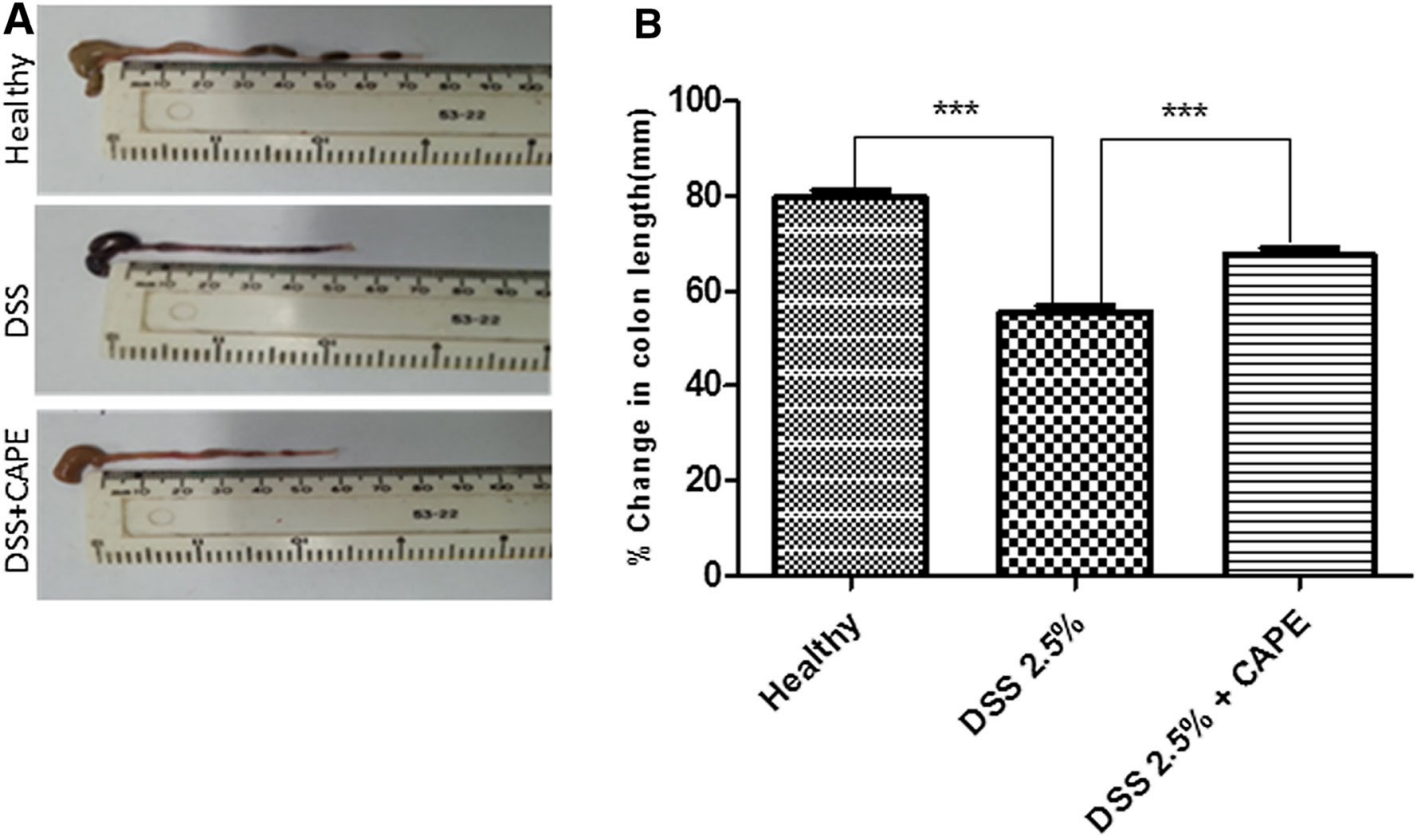
CAPE treatment is effective in protecting gross anatomy and colon length. **a** Gross appearance of the colonic anatomy shows the effect of CAPE on DSS-induced colon shortening and formation of fecal pellets. **b** Colon length was measured at post-mortem autopsy (*P* < 0.001). *N* = 5–6 mice per group

Shortening of the colon is one of the clinical signs of colitis (Tambuwala et al. 2015). Figure 3a shows representative image from the colon of a healthy mouse with well-formed stool pellets. In contrast, there were no formed stools and blood observed in the colon of mice treated with DSS alone. However, semi-formed stools and no blood were visible in the colon of mouse treated with CAPE. A graphical presentation of the average colon length of each group is shown in Fig. 3b. It was observed that there was significant (*P* < 0.001) reduction in colon length in mice treated with DSS alone when compared to the healthy control and DSS + CAPE-treated mice. Thus, CAPE treatment attenuated the impact of DSS on colon length reduction and also assisted stool formation.

**Fig. 4:**
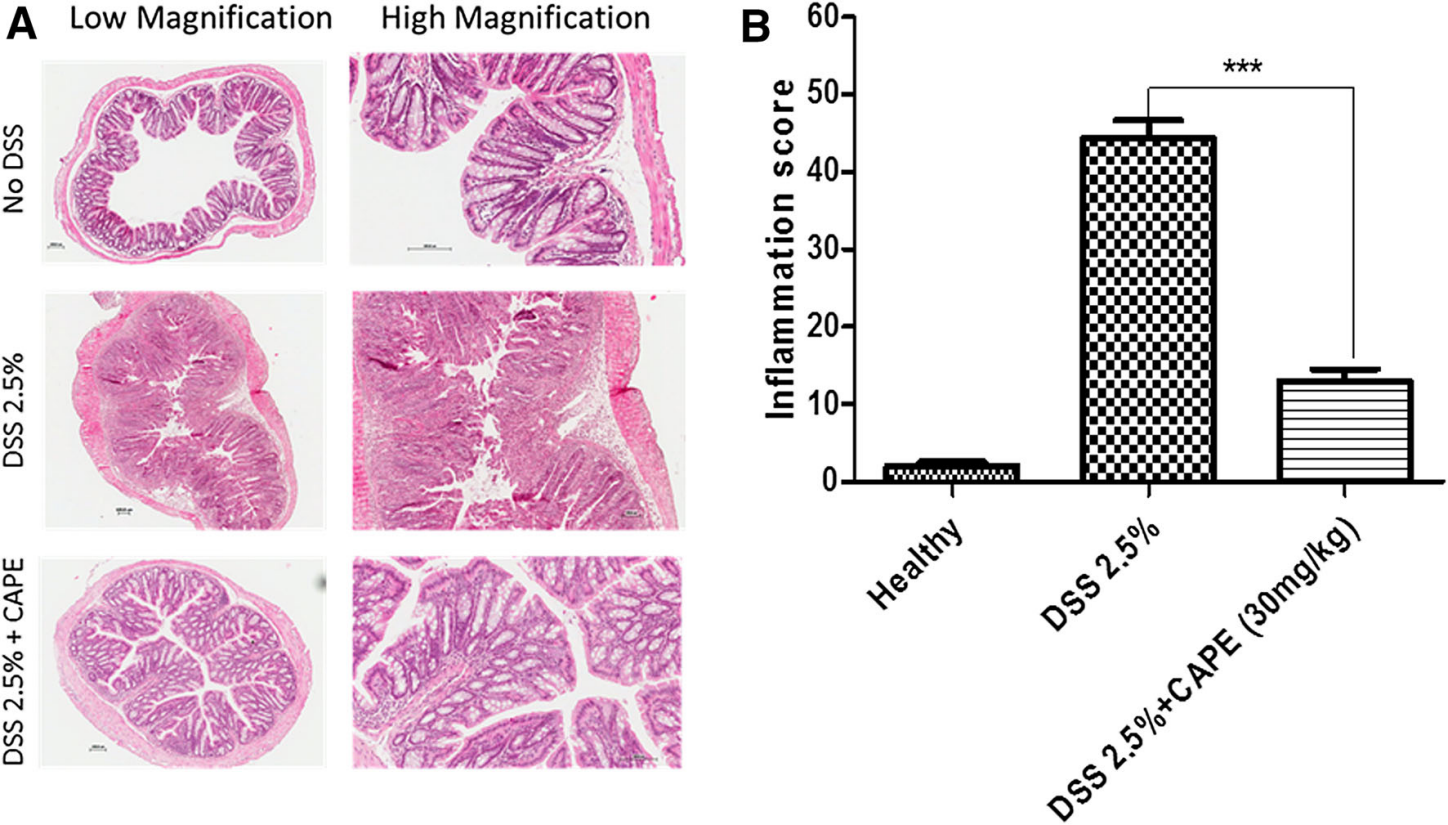
Improved colon histological outcome in mice treated with CAPE. **a** Representative histological images of colonic tissue showing the effect of CAPE treatment (H&E staining). **b** Histological scores of sections scored blinded. *N* = 5–6 mice per group (*P* < 0.001)

Histological examination of colon tissue confirmed that DSS treatment caused extensive colonic damage with lose of epithelium and collapse of crypt structure. This was accompanied by oedema and infiltration of inflammatory neutrophils (Fig. 4a). In contrast, there was a marked reduction in severity of DSS-induced colon injury in CAPE-treated mice. The crypt architecture showed that no ulceration or evidence of oedema, lesser degree of infiltration of inflammatory cells, and neutrophils were observed in the colon histology of mice receiving CAPE treatment (Fig. 4a). The blinded histological scoring of colon tissue histology revealed a significant reduction of damage in the colon of CAPE-treated mice relative to healthy control mice (*P* < 0.001; Fig. 4b). To confirm that CAPE downregulated the NF-κβ pathway in DSS-induced colitis, we assessed the levels of p65 in colon tissue. There was a marked increase in the level of p65 in DSS-alone group and the mice treated with CAPE showed a significant reduction in the level of p65 (Supplementary Figure 1).

**Fig. 5:**
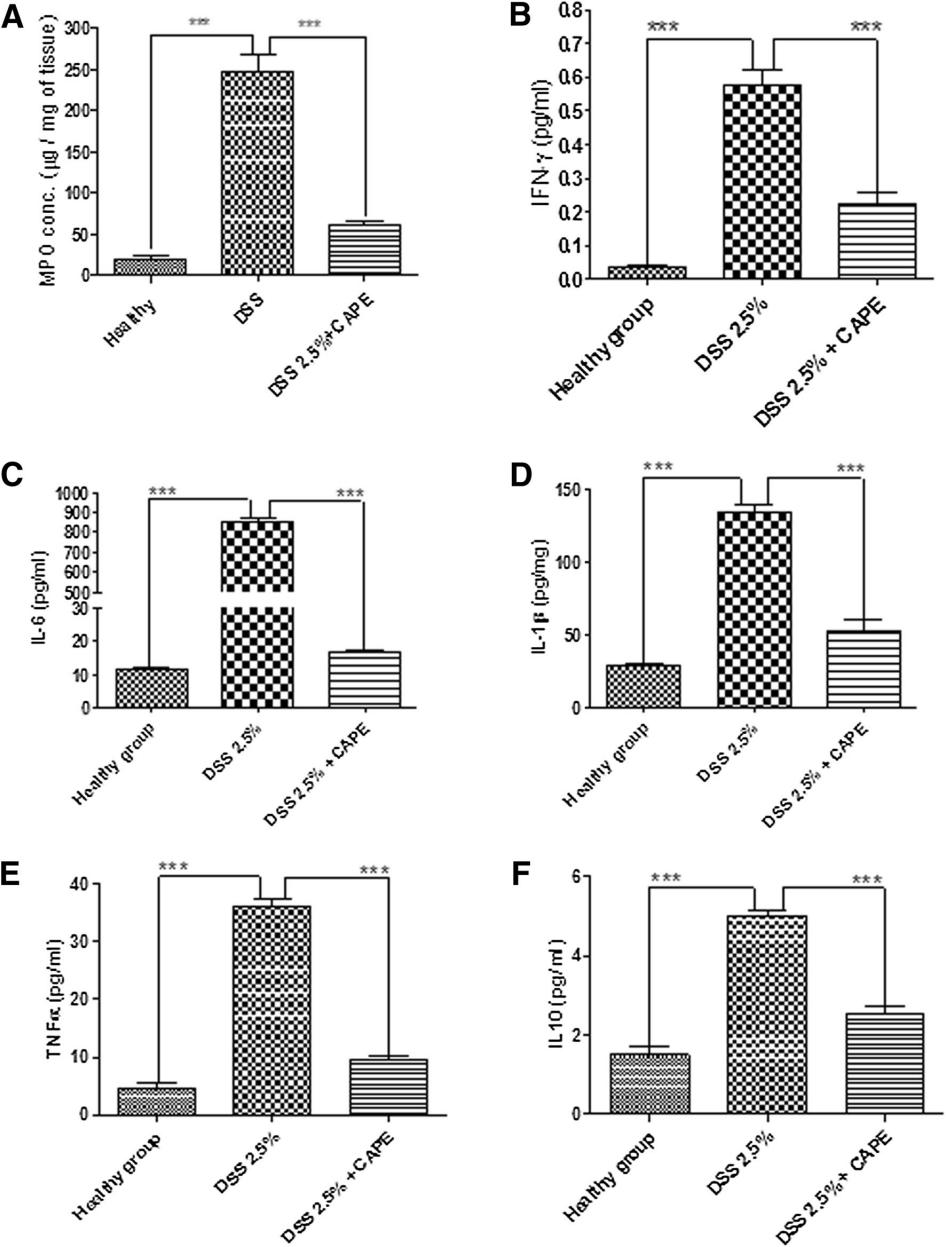
Effect of CAPE on expression of pro-inflammatory mediators. The colon tissue homogenates analysed for **a** MPO, **b** INF-ϒ, **c** IL-6, **d** IL-β, **e** TNF-α, and **f** IL-10. *N* = 5–6 mice per group (*P* < 0.001)

We next investigated the impact of CAPE treatment of the expression of markers of colonic inflammation that are increased in mice exposed to DSS. DSS-alone control mice showed a significant increase in MPO activity, a marker for inflammation, and leukocyte infiltration (*P* < 0.01; Fig. 5a). However, exposure of CAPE-treated mice to DSS did not result in increased colon MPO levels (Fig. 5a). We also noted that colonic levels of pro-inflammatory cytokines, such as INF-γ, IL6, IL1-β, TNF-α, and IL10 (Fig. 5b–e), were significantly (*P* < 0.001) increased in mice with DSS-induced colitis, as compared to healthy mice. Co-administration of CAPE resulted in small increases in INF-γ, IL1-β, TNF-α, and IL10, which were not significantly different from that of the healthy control. Thus, the DSS-induced colitis resulted in an increase in MPO, INF-γ, IL6, IL1-β, TNF-α, and IL10. All were diminished significantly in CAPE-treated mice (*P* < 0.01–0.001). Although IL-10 is known to play a protective role in colitis, we observed a small decrease in IL-10 levels in mice treated with CAPE, which was expected as CAPE is known to lower the levels of IL-10 (Sy et al. 2011). Furthermore, treatment of mice with CAPE alone had no effects on MPO, INF-γ, IL6, IL1-β, and TNF-α (data not shown).

### Enhanced epithelial barrier function in mice treated with CAPE

**Fig. 6:**
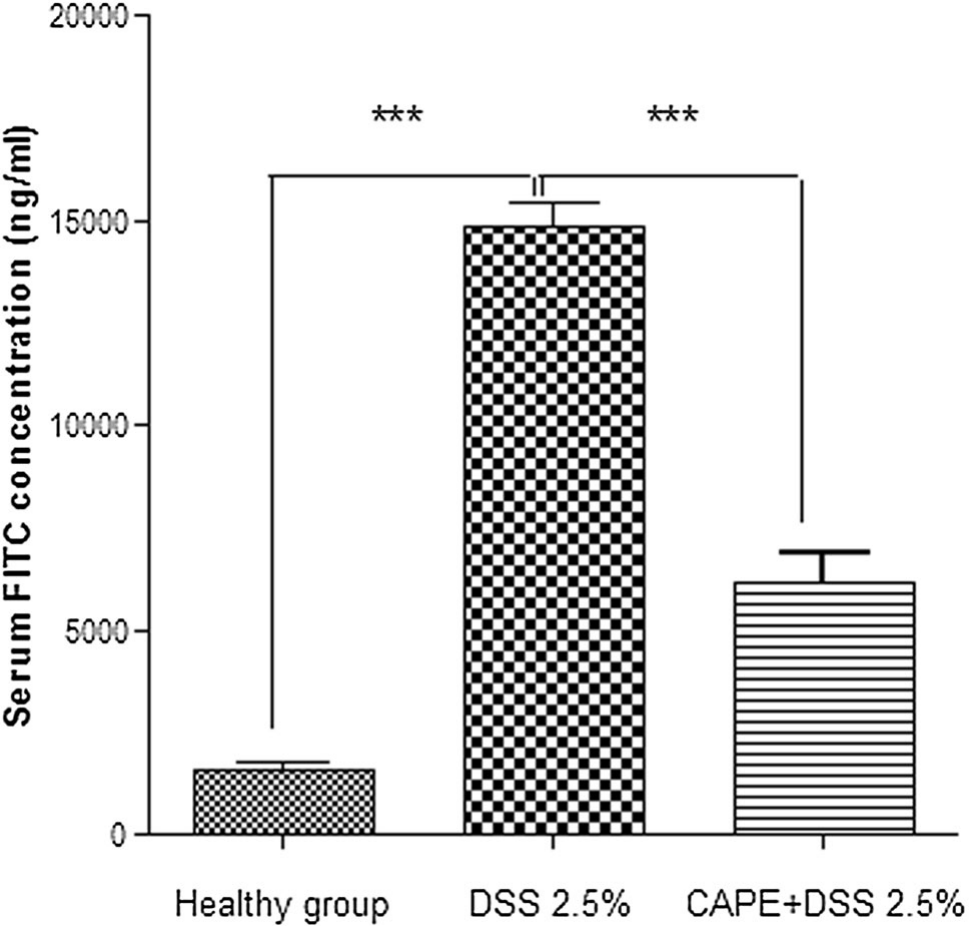
Reduced epithelial permeability in mice treated with CAPE. Mice treated with DSS with or without CAPE (30 mg/kg) IP and healthy mice were administered 4 kDa-FITC-labelled dextran orally, and serum levels of FITC were assessed. Each control and experimental group contains 5–6 mice (*P* < 0.001)

To investigate the effect of CAPE treatment on the intestinal epithelial integrity, in vivo barrier function was measured in healthy mice, mice exposed to DSS and mice co-treated with CAPE and DSS. An oral dose of FITC-dextran was administered to mice on the last day of DSS exposure. Four hours later, FITC levels in plasma were determined as a measure of intestinal permeability. The DSS-only group of mice exhibited a significant increase in intestinal permeability, which was reflected by an increased appearance of FITC in plasma. This effect was markedly diminished in mice treated with CAPE (Fig. 6a; *P* < 0.001), indicating that co-treatment with CAPE during DSS-induced colitis reduces the leakiness of the colon and maintains the epithelial barrier function.

## Discussion

The previous studies have indicated that CAPE is an effective inhibitor of NF-κβ and related cytokines in vitro, and also has the ability induce apoptosis in inflammatory cells (Fitzpatrick et al. 2001). In our current study, we have shown for the first time that CAPE significantly ameliorates the severity of the disease in a mouse model of UC.

One of the initial events that occur during the onset of IBD is disruption of the intestinal epithelial barrier function. This dysfunction leads to unwanted movement of luminal antigenic material into the *lamina propria*. This is followed by activation of mucosal immune cells and triggering of an inflammatory response. It has been suggested that one of the critical events in the development of inflammation in the intestine could be the regulation of intestinal epithelial cell apoptosis (Cummins et al. 2008; Tambuwala et al. 2010). In cases of chronic inflammation during IBD, constant intestinal epithelial cell apoptosis could lead the loss of the epithelial barrier, which will result in spread of inflammation, resulting in increased severity of the disease. This creates an imbalance in the innate and adaptive immunity of the gut (Nenci et al. 2007; Zaph et al. 2007).

In the present work, we have shown for the first time that CAPE, a potent inhibitor NF-κβ, is profoundly protective in an in vivo mouse model of acute colonic inflammation. Although we hypothesise that the protective effects of CAPE are mediated through the inhibition of the over activation of the NF-κβ pathway, we cannot exclude the possibility of NF-κβ independent mechanisms of action, such as inhibition of hydroxylases and activation of hypoxia inducible pathways (Cummins et al. 2008). However, several researchers have indicated that the NF-κβ pathway plays an important role during intestinal inflammation (Wei and Feng 2010; Buhrmann et al. 2011; Lawrence 2009a; Fitzpatrick et al. 2001) and inhibition of this pathway targets pro-inflammatory cytokines, such as interferons and tumour necrosis factor alpha. These are known to play key role during the development and progression of UC (Baird et al. 2016; Bishop et al. 2014; Ferrari et al. 2016).

CAPE treatment significantly ameliorated the severity of disease after acute DSS exposure in all parameters studied, including weight loss (Fig. 2a), clinical DAI score (Fig. 2b), reduction of colon length, and appearance of blood in faeces (Fig. 3a, b). A marked improvement in colon histology was observed (Fig. 4a), together with improved blinded inflammation scores (Fig. 4b). In the murine model of DSS-induced colitis, the increase in MPO and pro-inflammatory cytokines occurred after the disruption to the intestinal barrier, indicating that compromised barrier function results in progression inflammation. CAPE-treated mice did not have increased MPO (Fig. 5a) and only small increase in other pro-inflammatory cytokines (Fig. 5b–f), suggesting that CAPE treatment prevented the damage to colon epithelial cells caused by DSS and helps in maintaining the epithelial barrier function, which is evident by reduced permeability of FITC in mice treated with CAPE (Fig. 6). However, whether improved epithelial barrier function or indeed lowered cytokine expression is the cause or consequence of the protective effects of CAPE remains to be elucidated. This critical question will be the topic of further investigations.

In mice treated with CAPE, in the absence of DSS exposure, there were no alterations in MPO or cytokines in the colon and no alterations in colon histology or length. This confirms that in the acute 6-day treatment regimen used in this study, CAPE did not alter physiologic inflammation in normal tissue, but instead suppressed inflammation in the colon when occurred due to DSS-induced disruption of the barrier function.

In this study, we have observed that there was an increase in NF-κβ activity in the colon of mice treated with DSS and that CAPE downregulates this increase, thereby exerting a protective event in a mouse model of UC. Since CAPE is a natural compound, with no known side effects (Tambuwala 2016), its therapeutic benefits are obvious and desirable when measured up against over other novel compounds with pro-tumorigenic effects; such as DMOG, which have also shown to be protective in experimental colitis (Cummins et al. 2008). The findings of this work indicate that CAPE can be used an effective first-line treatment for patients with UC, improving intestinal barrier function and halting the progression of disease, whilst promoting mucosal healing. The next stage of our work will focus on two elements, namely (1) development of nanoparticle-based colonic drug delivery of CAPE, which could allow for local delivery of the drug to inflamed tissue to ensure effective therapeutic outcomes using a lower dose and (2) identification of the NF-κβ subunit most affected by CAPE.

## Electronic supplementary material

Below is the link to the electronic supplementary material.

**Supplementary Figure 1. Assessment of** ***NF*** **-** ***κβ/p65 levels in colon tissue.*** The colon tissue extracts evaluated for NF-κβ/p65 levels in healthy, DSS 2.5% and DSS 2.5% + CAPE. N = 5–6 mice per group (P < 0.001). (TIFF 763 kb)
