# Supplementary figures and images for: Caffeic acid phenethyl ester is protective in experimental ulcerative colitis via reduction in levels of pro-inflammatory mediators and enhancement of epithelial barrier function
Source: Inflammopharmacology. 2017 May 20;26(2):561–9. doi: 10.1007/s10787-017-0364-x (PMC5859149; doi:10.1007/s10787-017-0364-x)

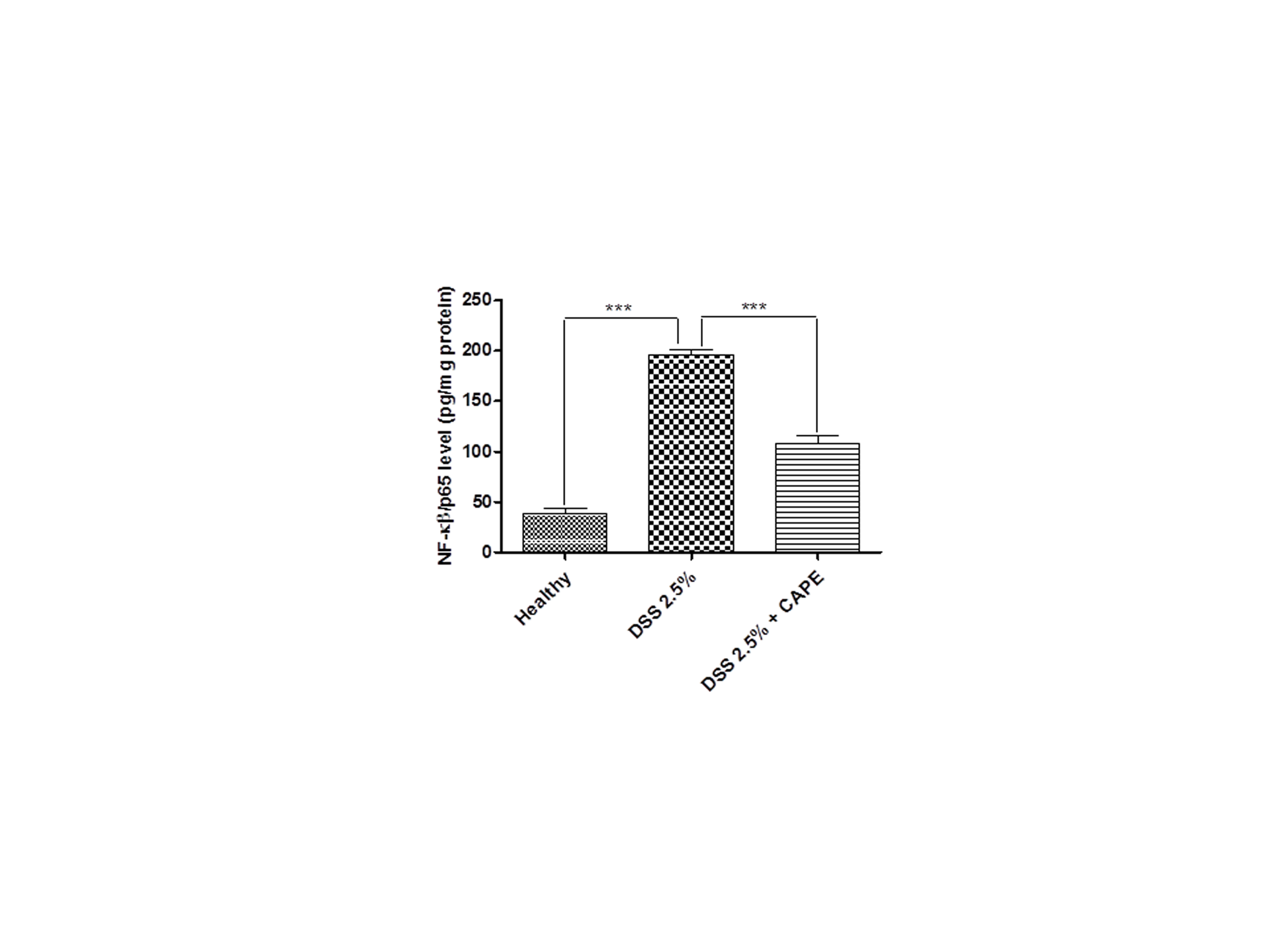

Supplement: Supplementary file 1 — Supplementary Figure 1. Assessment of NF - κβ/p65 levels in colon tissue. The colon tissue extracts evaluated for NF-κβ/p65 levels in healthy, DSS 2.5% and DSS 2.5% + CAPE. N = 5–6 mice per group (P < 0.001). (TIFF 763 kb) [file 10787_2017_364_MOESM1_ESM.tif]
